# Efficient Laser-Driven Proton Acceleration from a Cryogenic Solid Hydrogen Target

**DOI:** 10.1038/s41598-019-52919-7

**Published:** 2019-11-11

**Authors:** J. Polz, A. P. L. Robinson, A. Kalinin, G. A. Becker, R. A. Costa Fraga, M. Hellwing, M. Hornung, S. Keppler, A. Kessler, D. Klöpfel, H. Liebetrau, F. Schorcht, J. Hein, M. Zepf, R. E. Grisenti, M. C. Kaluza

**Affiliations:** 1 Institut für Optik und Quantenelektronik, Friedrich-Schiller-Universität, 07743 Jena, Germany; 2 Central Laser Facility, Rutherford-Appleton Laboratory, Chilton, Oxon, OX11 0QX UK; 3 Institut für Kernphysik, J. W. Goethe-Universität, 60438 Frankfurt a.M., Germany; 4 Helmholtz-Institut Jena, 07743 Jena, Germany; 5 Department of Physics and Astronomy, Queen’s University Belfast, Belfast, BT7 1NN UK; 6 GSI - Helmholtzzentrum für Schwerionenforschung mbH, Planckstr. 1, 64291 Darmstadt, Germany

**Keywords:** Laser-produced plasmas, Plasma-based accelerators

## Abstract

We report on the successful implementation and characterization of a cryogenic solid hydrogen target in experiments on high-power laser-driven proton acceleration. When irradiating a solid hydrogen filament of 10 μm diameter with 10-Terawatt laser pulses of 2.5 J energy, protons with kinetic energies in excess of 20 MeV exhibiting non-thermal features in their spectrum were observed. The protons were emitted into a large solid angle reaching a total conversion efficiency of several percent. Two-dimensional particle-in-cell simulations confirm our results indicating that the spectral modulations are caused by collisionless shocks launched from the surface of the the high-density filament into a low-density corona surrounding the target. The use of solid hydrogen targets may significantly improve the prospects of laser-accelerated proton pulses for future applications.

## Introduction

Since the first observation of Mega-electronvolt (MeV) proton beams produced during the interaction of high-intensity laser pulses with thin foils^1,2^, the field of laser-ion acceleration has seen rapid progress. Quasi-static, Teravolt-per-meter (TV/m) electric fields are generated during these interactions, orders of magnitude higher than those achievable with conventional accelerators. Due to the short acceleration length (∼10 *μ*m) required for protons to reach energies of several tens of MeV in these fields, various potential applications such as the production of medically relevant radio-isotopes^3^, hadron therapy^4,5^, or the realization of an ultra-short frontend for conventional accelerators^6–8^ are under discussion. These applications would all benefit from the availability of an intense and compact high-energy proton source, preferably with a high repetition rate. However, broad, quasi-thermal energy distributions of the protons and a low energy-conversion efficiency from the laser to the proton beam have been major issues in the past, which need to be solved before the envisaged applications can be realized.

MeV-protons can be generated via the process of Target Normal Sheath Acceleration (TNSA)^9^. Here, a high-intensity laser pulse ionizes the front side of a solid target, e.g. a thin foil, generating hot electrons. These electrons propagate through the target to form a sheath at its back side. Rear-surface atoms are first ionized and then accelerated away from the target by the associated TV/m-electric fields. While *μ*m thick metal targets are commonly used, protons stemming from surface contaminations are favored due to their highest charge-to-mass ratio, *q/m*, hence dominating the acceleration. Nevertheless, a significant fraction of the laser energy is distributed among heavier ions^10^ reducing the energy conversion from laser to protons. For high-repetition rate lasers with sub-100 fs pulse duration and 10–200 TW peak power, this efficiency has been reported to be 1% or less^11^. So far, only single-shot, PW-class lasers with pulse energies of a few 100 J have reached conversion efficiencies of 6% with a proton-beam half-angle of ∼30°^12^. Furthermore, using the PW-laser system VULCAN, conversion efficiencies as high as 15% from laser energy to protons could be achieved when using thin Au-foil targets irradiated by a double-pulse structure^13^. Finally, TNSA protons typically exhibit a Boltzmann-like spectrum^14^, but can be modified by specially prepared targets^15,16^.

Another process producing MeV protons is Radiation Pressure Acceleration (RPA)^17,18^. Here, the laser pulse is reflected at the target’s front side pushing the electrons in the forward direction, i.e. into the target. The negatively charged electrons in turn pull along the positive ions. RPA requires balancing the pressure from laser radiation with the pressure from charge separation in the target. This is primarily achievable with nm-thin solid targets^19,20^. Yet another possible mechanism is the acceleration of ions by collisionless electrostatic shocks (Collisionless Shock Acceleration, CSA). Such shocks can, e.g., be generated at a sharp transition from a hot, dense plasma to a cooler plasma of lower density^21^. A strong electric field spike is formed at the shock front, accelerating ions from the less dense plasma that the shock propagates into.

The problem that a considerable fraction of the energy is imparted to heavier ions occurs in all of these mechanisms. Hence, there is good reason to consider pure hydrogen targets, which can readily be produced e.g. with gas jets, ensuring that protons are the only accelerated ion species. However, both RPA and shock acceleration demand that an over-critical plasma be generated. For regular-pressure gas jets, long-wavelength lasers such as CO_2_-lasers are required. Here, narrow-band proton beams have been observed^22,23^ but with low conversion efficiencies (4 × 10^−4^). If near-IR, high-power lasers are to be used, they require either the use of ultra-high pressure gas jets^24,25^ or hydrogen targets at near-solid density^26–28^. Furthermore, a self-replenishing target, which is well-suited for high-repetition rate operation would be beneficial. Over the last few years, there has been considerable research on the application of solid-hydrogen as the target material. The results from these measurements show a high conversion efficiency from laser energy to protons when using 300 ps-long^26^ or sub-ps pulses^28,29^. In most of these experiments, temperature-like proton spectra following a Boltzmann distribution were detected with cutoff-energies in the range of 1 MeV^26^ or up to 20 MeV^28^. In the results presented by Gauthier *et al*.^27^, a quasi-monoenergetic feature around 1 MeV was observed but the exact origin of this feature has not yet been identified. Furthermore, Göde *et al*. found that due to the presence of a preplasma on the target rear surface, Weibel-type instabilities affecting the formation of the hot-electron sheath on the target rear surface can strongly modulate the generated beam profile in the transverse direction^30^, rendering such beams problematic for applications, which require a smooth proton beam.

In this paper, we report the successful application of a cryogenic solid hydrogen target^31,32^ for laser-driven proton acceleration. Using a Joule-class, 1/40-Hz laser system, we achieved both a high energy conversion efficiency from the laser pulse to the accelerated proton beam and a cutoff-energy in excess of 20 MeV while still exhibiting a rather smooth beam profile. Furthermore, we observed clear non-thermal features in the proton spectra, which can be explained to be the result of an electro-static, collisionless shock occurring in the low-density corona surrounding the solid-hydrogen filaments, therefore offering a new explanation for our – and potentially also for other – experimental results.

## Experimental Setup

**Figure 1:**
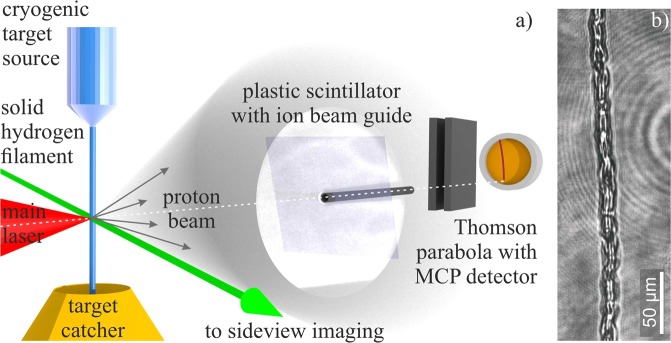
Experimental setup. (**a**) Schematic of the experimental setup in the evacuated interaction chamber. Here, laser pulses (red) from the POLARIS system irradiate the solid hydrogen filament (blue) vertically emitted from the cryogenic target source. Before the interaction, the filament’s position with respect to the focal plane of the laser can be controlled with a sideview-imaging system using a frequency-doubled probe laser pulse from a Nd:YAG laser with ns duration (green). Protons emitted during the interaction (grey) are first detected by a plastic scintillator. A gateable CCD camera (not shown here), which is looking at this scintillator from the back, provides energy-resolved information about the proton beam’s spatial profile. Through a hole in the scintillator and an ion beam guide aligned to the laser forward direction protons can propagate towards a Thomson-parabola ion spectrometer with parallel electric and magnetic fields equipped with a micro-channel plate (MCP) as the detector. With this spectrometer, energy spectra of the protons and any other ion species could be detected. (**b**) Sideview image of the solid hydrogen filament around the laser focus position but without the main pulse.

In a cryogenic microjet source, 99.999% purity hydrogen was pressurized up to 30 bar and liquefied with a continuous flow liquid helium cryostat at a working temperature of 14 K to 19 K. Through a (10 ± 0.5)*μ*m diameter glass capillary nozzle the liquid was injected with a laminar flow into the evacuated main interaction chamber, emerging as a continuous cylindrical stream with a diameter set by the nozzle’s cross section. The propagating liquid then rapidly cooled by surface evaporation until it froze producing a continuously replenishing solid filament^31^, cf. Fig. 1. While the hydrogen target solidifies before it is irradiated by the high-intensity laser pulse, the formation of a contamination layer on its surface, which might contain other ion species as commonly present in standard solid-target interactions, is nevertheless suppressed. The liquid hydrogen jet is emitted at a speed of ∼166 m/s from the nozzle and the filament is irradiated by a high-intensity laser pulse at a distance of 13 mm below the orifice (see next paragraph). The corresponding propagation time of ∼78*μ*s might be sufficient to adsorb e.g. hydro-carbon contaminants from the rest-gas in the interaction chamber, in particular when the low temperature of the filament is considered. However, the vapor pressure of solid hydrogen at this temperature leads to the formation of a low-density corona of hydrogen gas surrounding the filament. During this evaporative process (which is responsible for the freezing of the liquid hydrogen in the first place) any adsorbed contaminants would immediately be blown away again. We can therefore assume that the target consists of pure hydrogen only. This assumption is also confirmed by the fact that with the Thomson parabola spectrometer, which was used for the detection of the accelerated ions (see below), only protons and no other ion species were measured.

At a distance of 13 mm below the nozzle’s orifice, the filament was irradiated by 1030 nm linearly polarized pulses of 2.5 J energy and 217 fs duration (intensity FWHM) from the POLARIS laser system^33^. The pulses were focused by an off-axis parabolic mirror (*F*_*#*_ = 2.5) onto the filament under normal incidence. In a focal spot area of 8.4 μm^2^, 25.9% of the laser pulse energy was contained, resulting in an on-target intensity of 3.5 × 10^19^ Wcm^−2^ corresponding to a normalized vector potential of ${a}_{0}=8.55\times {10}^{-10}{\lambda }_{{\rm{L}}}(\mu {\rm{m}})\times \sqrt{{I}_{{\rm{L}}}({\rm{W}}c{m}^{-2})}\approx 5.2$. Using an optical probe pulse of ns duration, the position of the hydrogen filament could be aligned and monitored with respect to the focal plane of the laser before the interaction, cf. Fig. 1b). The standard deviation of the filament’s front surface from the focal plane was 4.0 μm.

In the laser’s forward direction, a 5 mm thick, fast responding plastic scintillator [Saint Gobain BC-422Q, signal pulse width 360 ps (FWHM) and signal decay time 700 ps] detected the proton beam profile. Positioned 575 mm behind the target, it covered a solid angle of 72.1 msr, the half-opening angle in the horizontal direction was 9.4°. The scintillator was light-shielded by 30 μm of aluminum and imaged onto a gateable CCD with a minimal gate ≤1 ns. The time of flight (TOF) from target to scintillator was 1.9 ns for γ–rays and MeV electrons and ≥9.3 ns for ≤20 MeV protons. This difference was sufficient for a clear distinction between these particles on the scintillator. By an appropriate choice of the gate’s width and delay with respect to the main pulse we could record the proton beam profile for energies between 3 and 20 MeV^34^. Through a hole in the scintillator aligned to the laser axis, protons could propagate towards a Thomson parabola spectrometer, covering a solid angle of 1.07 *μ*sr. In this spectrometer, the protons were dispersed by parallel magnetic (ℬ ≈ 600 mT) and electric fields (ℰ = 750 kVm^−1^) and then detected by a micro channel plate (MCP), which had been absolutely calibrated using CR39 nuclear track detectors^35^. At the lower cut-off energy of 3 MeV determined by the MCP’s size, the energy resolution was Δ*E* = 200 keV.

## Experimental Results

**Figure 2:**
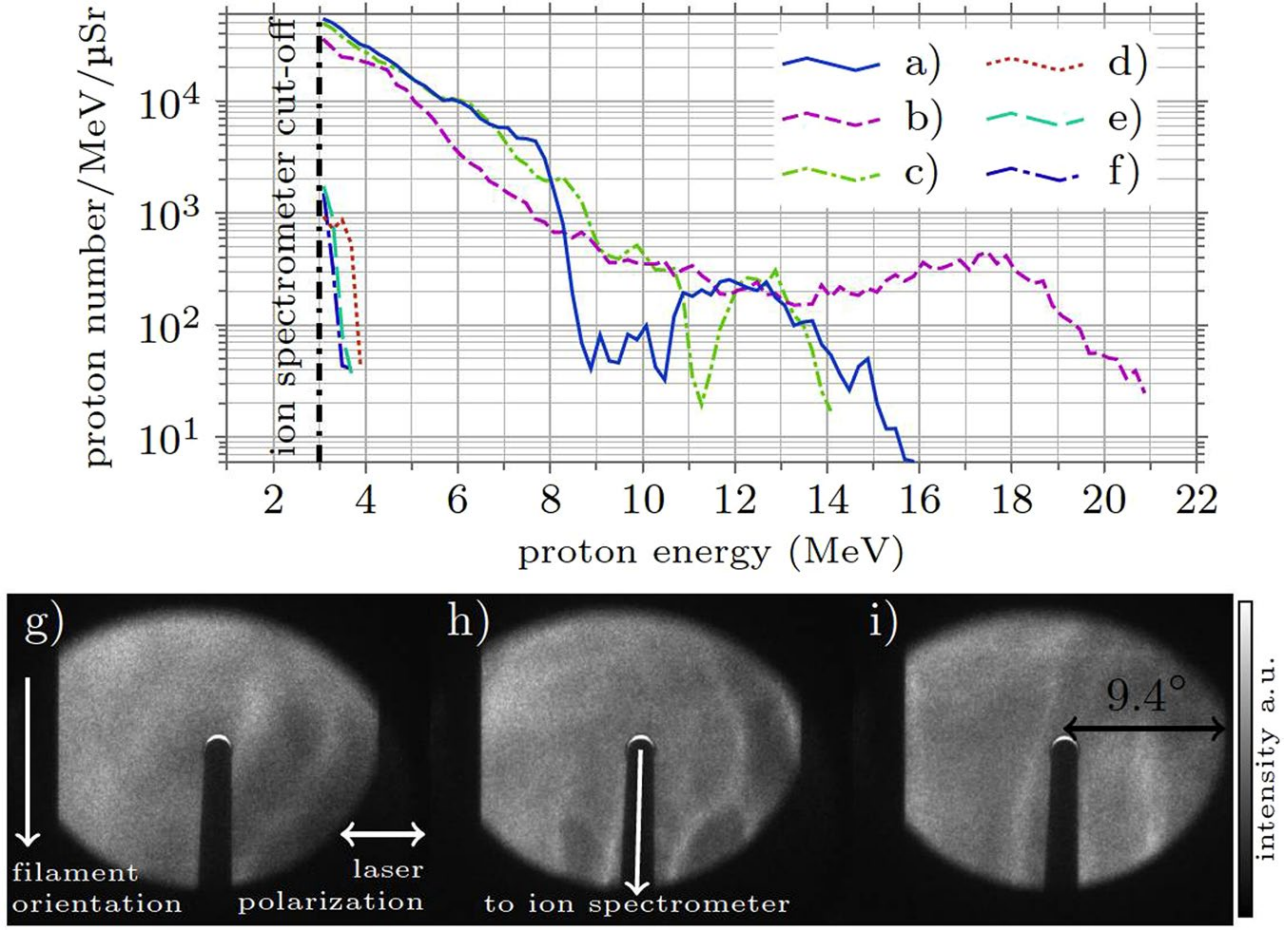
Experimental results I. Proton energy spectra (**a–f**) and beam profiles (**g–i**). The spectra (**a–c**) and the corresponding beam profiles (**g–i**) were obtained with a temporal intensity contrast TIC = 3.6 × 10^−9^ at a time 30 ps before the main pulse (for a definition of the TIC see text). While the low-energy part of the spectrum in (**a–c**) shows an exponential decay, modulations are visible at higher energies. The scintillator images (9.4° half-opening angle) show beam profiles with no clear intensity drop towards the edges of the field of view, indicating an emission of protons into a significantly larger opening angle. Note that the scintillator’s rear side is imaged onto the CCD. The black shadow visible in the images of the beam profiles is due to the tube used as the ion beam guide towards the spectrometer, which blocks part of the image. The spectra (**d–f**) correspond to shots with TIC = 4 × 10^−8^. Here, only 5 out of 81 shots produced protons with energies only slightly above the spectrometer’s lower cut-off of 3 MeV.

The description of the reported results is based on a set of 2197 shots. Influenced by the stability of the filament, proton spectra exceeding the lower cut-off energy of the spectrometer (3 MeV) were recorded in 65.4% of all shots. In 30.5% of our recorded spectra the low-energy part showed an exponential decay as expected by TNSA, but the high-energy part of the spectrum (i.e. above ≈8 MeV extending up to 21 MeV) exhibited clear non-thermal features. Three exemplary proton spectra showing such modulations are shown in Fig. 2a–c).

No other ion species were detected. Simultaneously, we recorded the proton beam profiles with the scintillator. The profiles in Fig. 2g–i) correspond to the spectra a)-c). Here, no clear lateral decay of the signal could be detected in our field of view indicating a proton emission into a significantly larger opening angle than covered by the scintillator. Furthermore, we observed no or only low-amplitude high-frequency spatial modulations^36^, which would have hinted towards plasma instabilities occurring during the acceleration process or the subsequent propagation^30^. Such spatial instabilities would likely lead to spectral variations, too. We therefore assume that the proton spectrum emitted in different directions only slowly varies. The conversion efficiency from laser to protons with 3 MeV ≤ *E *≤ 20 MeV emitted into the solid angle covered by the scintillator was between 0.3% and 0.9%.

Earlier simulations^37^ found that when using *μ*m-thin foils as a target, non-thermal features in the proton spectra are only generated when the acceleration is limited to a spatially confined source containing another ion species with lower *q/m*. In comparison, our target was a virtually infinitely long cylinder of pure hydrogen that nevertheless produced spectral modulations. However, the solid hydrogen filament cannot be assumed to exhibit a step-like density profile at its surface. At the triple point of hydrogen (71.9 mbar and 13.947 K^38^), the vapor pressure leads to the formation of a gas corona surrounding the filament. Close to the surface, the gas density is equivalent to *n*_e_ = 7.6 × 10^19^ cm^−3^ = 0.072*n*_*c*_ (for a critical density ${n}_{{\rm{c}}}=4{\pi }^{2}{\varepsilon }_{0}{m}_{{\rm{e}}}{c}^{2}/({\lambda }_{{\rm{L}}}^{2}{e}^{2})$ for λ_L_ = 1030 nm). Therefore the target consists of a core of solid hydrogen with 10 *μ*m diameter, which will be ionized to *n*_*e*_ = 49.3*n*_*c*_, surrounded by a corona with a density almost three orders of magnitudes smaller. Assuming a stationary isothermal expansion of the evaporating gas and a vapor density *n*_0_ at the filament’s surface at *r*_0_, the corona density scales according to Fick’s law of diffusion as $n(r)={n}_{0}/[3\pi {d}_{{{\rm{H}}}_{2}}^{2}{n}_{0}(r-{r}_{0})+1],$ where$\,{d}_{{{\rm{H}}}_{2}}$ is the molecular radius of hydrogen. This leads to a slow rarefaction of the corona close to the target surface.

**Figure 3:**
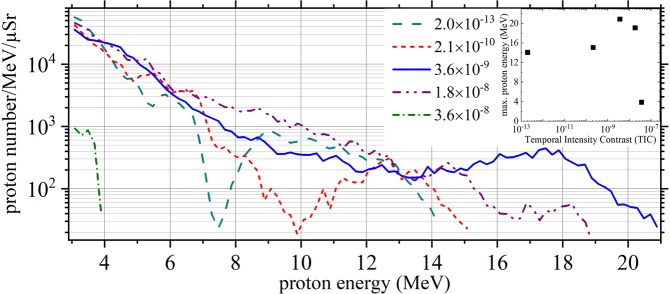
Experimental results II. Proton energy spectra for five different values of the temporal intensity contrast (TIC). The small inset shows the maximum proton energy depending on the TIC value for these five shots.

To investigate the possible influence of the laser prepulse on the acceleration process, the laser’s temporal intensity contrast (TIC) due to amplified spontaneous emission (ASE) was modified. To accomplish that, two alternative frontends could be used^39^, intrinsically generating seed pulses for the final amplifiers with different initial TIC. Furthermore, we could reduce the seed energy for one of the amplifiers in the  POLARIS chain and simultaneously increase its gain. As a result of these measures, the TIC at a time 30 ps before the main pulse could be varied between *I*_ASE_/*I*_0_ = 2 × 10^−13^ and 4 × 10^−8^, while keeping the main pulse energy and duration constant^39,40^. The generation of modulated proton spectra was observed over a wide range of TIC (2 × 10^−13^ < *I*_ASE_/*I*_0_ < 2 × 10^−8^). In this TIC range the achievable proton cut-off energies were extending up to 14–21 MeV, cf. Fig. 2a–c) and Fig. 3. Nevertheless, shot to shot fluctuations of the cut-off energy occurred even for fixed laser parameters. These fluctuations can likely be attributed to the spatial instability of the filament. A number of exemplary proton energy spectra as measured with the Thomson parabola for different values of the TIC are shown in Fig. 3. When worsening the pulse contrast further, the energy of the accelerated protons immediately dropped close to or below 3 MeV, the low-energy limit of the spectrometer, cf. Fig. 2d–f) and the small inset of Fig. 3, where the maximum proton energy depending on the TIC is shown. It is likely that in this case the ASE prepulse significantly changed the target characteristics before the main interaction, rendering the ion acceleration ineffective. We therefore conclude that for the acceleration of protons from solid-hydrogen filaments showing non-thermal features in their energy spectrum, any prepulse-induced plasma expansion of the solid filament prior to the interaction with the main pulse plays a subordinate role only – as long as *I*_ASE_/*I*_0_ < 2 × 10^−8^. It is more likely that the corona surrounding the filament is the reason for the observed spectral modulations.

## Numerical Simulations

To study the influence of the low-density corona surrounding the filament we performed two-dimensional particle-in-cell (2D-PIC) simulations using the Osiris code^41^. These simulations were performed using a 12000 × 12000 grid with a total size of 400 × 400 (*c*/ω_*L*_)^2^. The target consisted of a circular filament of purely hydrogenic plasma centered at (100, 200) (*c*/ω_*L*_)^2^ with a radius of 32 *c*/ω_*L*_ and an electron/proton density of 40 *n*_*c*_. The filament in turn was surrounded by a circular low-density corona that extended out another 12.5 *c*/ω_*L*_ in radius. Due to the slow rarefaction of the evaporating hydrogen close to the filament surface it was assumed in the simulation to have a uniform density of 0.08 *n*_*c*_. A linearly polarized laser pulse was incident on this target from the left hand boundary along the line *y* = 200 *c*/ω_*L*_. The pulse had a triangular temporal profile with rise/fall times of 377 ${\omega }_{{\rm{L}}}^{-1}$, a focal spot radius of 12 *c*/ω_*L*_, and *a*_0_ = 5.2.

**Figure 4:**
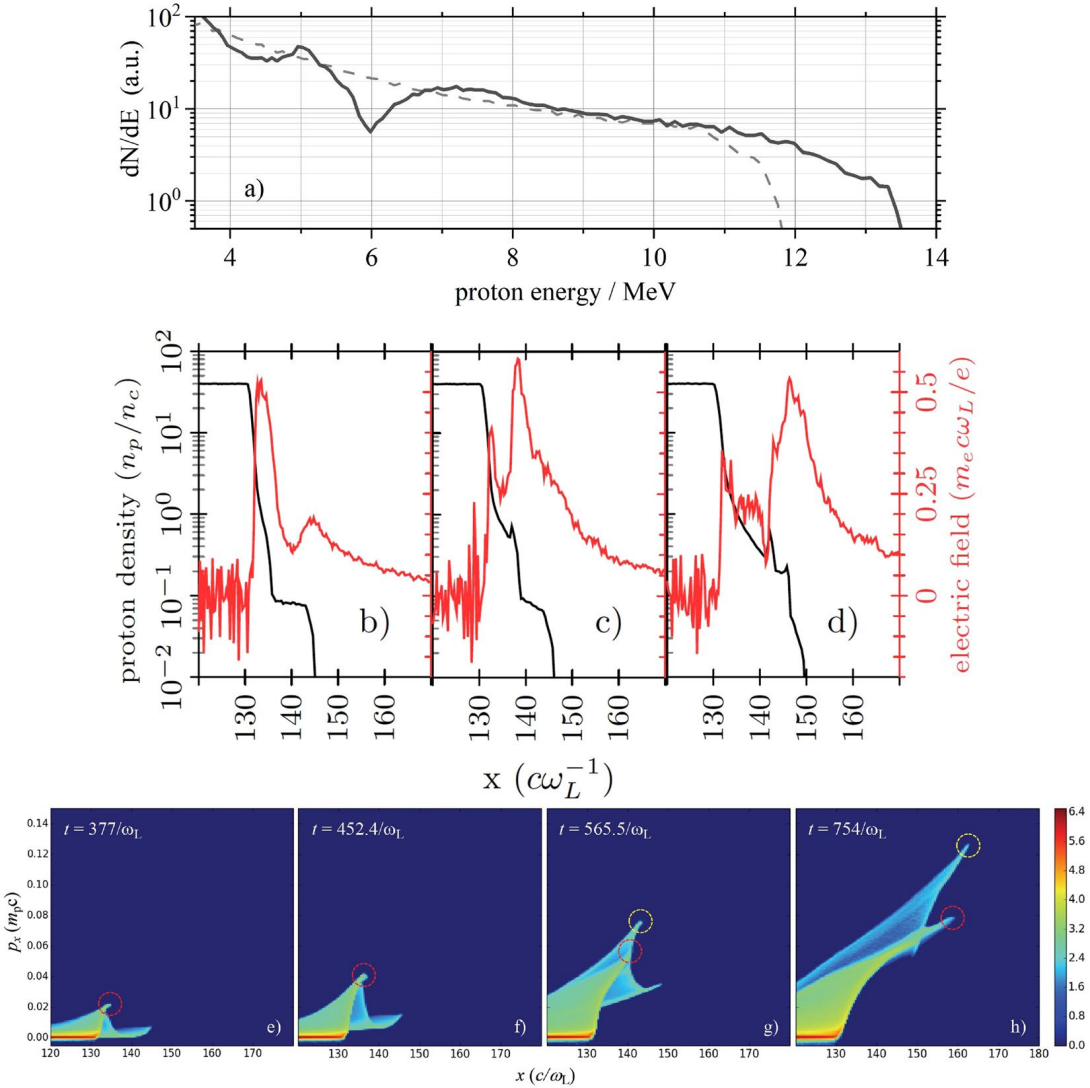
Numerical results I. (**a**) Simulated proton energy spectra within an opening angle of 1 mrad around laser forward direction from the simulation at $t=1508\,{\omega }_{{\rm{L}}}^{-1}$. The solid black line shows the spectrum for the case with a low-density corona surrounding the solid hydrogen filament, while the grey dashed line corresponds to the spectrum without the corona. (**b–d**) Plots showing both the electric field (red, right abscissa, normalized to *m*_*e*_*c*ω_*L*_/*e*) and proton density (black, left abscissa, normalized to the critical density *n*_*c*_) at times $377\,{\omega }_{{\rm{L}}}^{-1}$ (**b**), $452.4\,{\omega }_{{\rm{L}}}^{-1}$ (**c**) and $565.5\,{\omega }_{{\rm{L}}}^{-1}$ (**d**) at the filament’s rear side. Here, *n*_*c*_ ≈ 1.05 × 10^21^/cm^2^ and *m*_*e*_*c*ω_*L*_/*e* ≈ 3.12 TV/m for λ_L_ = 1.03 *μ*m. (**e–h**) Protons’ phase space at the filament’s rear side showing the acceleration process in detail. At the first time step (**e**), two TNSA-type acceleration regions can be distinguished, one at the transition from the solid-density surface to the low-density corona (around *x* = 133 *c*/ω_L_) and another one at the corona-vacuum boundary (at *x* = 144 *c*/ω_L_). The shock acceleration occurs at the position, where the protons, which have been accelerated via TNSA from the filament’s solid-density surface move into the low-density region of the corona, where background protons are accelerated to a significantly higher velocity than the velocity of the piston due to the reflection off this shock front. The fastest protons accelerated by TNSA from the solid-corona boundary are indicated in all four images by the dashed red circles, while the fastest protons, which have been accelerated by reflection at the shock front and which can be clearly identified at the last two time steps, are indicated by the dashed yellow circles.

The main result of this simulation can be seen in a plot of the proton energy spectrum from *t* =  1508 ${\omega }_{{\rm{L}}}^{-1}$, which is shown by the solid black line in Fig. 4a. Only protons traveling at an angle of less than 1 mrad with respect to the laser-axis contribute to this spectrum. It clearly contains non-thermal features that are not dissimilar to those observed in the experiment. In particular, the spectrum shows a deep cleft and a pronounced peak in what would otherwise be described as a ‘thermal’ distribution. It is important to point out that this only occurred in simulations including the low-density corona. In a simulation without the corona these features were no longer present as can be seen by the dashed grey line in Fig. 4a. Hence our simulations support our interpretation that the corona is essential to the generation of spectral modulations, as they are also seen in our experimental results.

## Discussion

We characterize the process producing these spectral features as being ‘collisionless shock acceleration^21,23,42^. Overall, the protons are accelerated by TNSA from the filament’s rear surface, i.e. by a sheath field generated by hot electrons, hence the overall thermal energy spectrum. The exact shape and extension of the corona will slightly affect the amplitude of this sheath field, which is likely to explain the slight difference in peak energy between experiment and simulations. The presence of the corona additionally affects the electric field structure, cf. Fig. 4b–d. In addition to the sheath field at the corona-vacuum boundary, one also gets a second, initially higher electric field spike forming at the interface between the high-density core of the target and the low-density corona. This is a natural consequence of having a change in the ion density profile over a scale length, which is much shorter than the Debye length λ_D_ = [*ε*_0_*k*_B_*T*_e_/(*n*_eh_*e*^2^)]^1/2^ associated with the hot electrons (which we estimate to be in excess of 0.3 *μ*m).

This spike leads to the formation of a collisionless electrostatic shock that propagates into the corona region. The spike moves together with the step-like change in the proton density, cf. Fig. 4b–d), which can also be seen in the evolution of the protons’ phase space as shown in Fig. 4e–h). Since initially the strongest acceleration of protons occurs in the locality of this shock, protons will experience a ‘pistoning’ type of acceleration on encountering this shock. Piston-type acceleration like this will produce a monoenergetic bunch if the drive is uniform and constant in nature and if the bunch undergoes no further acceleration. In our case, the drive is neither strictly constant nor strictly uniform, and the protons accelerated by this undergo further acceleration. Nonetheless, this effect is sufficient to produce a distinct cleft in the spectrum and a slight peak at higher energies as also observed in the experiment.

**Figure 5:**
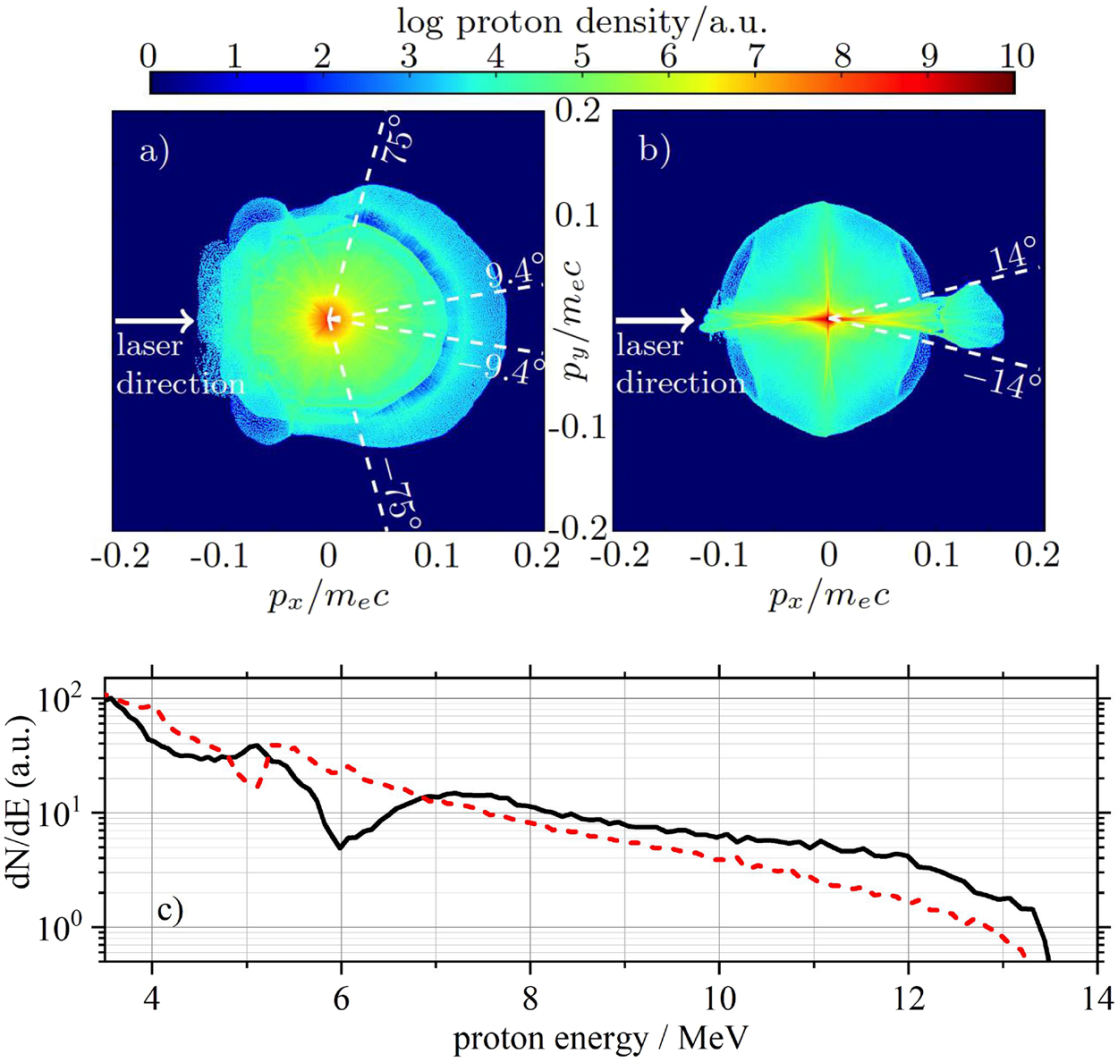
Numerical results II. Simulated proton phase space at $t=1131\,{\omega }_{{\rm{L}}}^{-1}$ with (**a**) cylindrical and (**b**) planar targets. (**a**) The half-ring like region in the right part indicative of strong modulations in the proton numbers, represents the modulation seen in the energy spectra. These features are present and uniform over a large emission angle of ±75°. The scintillator’s acceptance angle (±9.4°) is also shown. (**b**) Simulation using a rectangular target (1 × 20 μm^2^) showing a significant reduction in emission angle to ±14°. (**c**) Simulated proton energy spectra within an opening angle of 01 mrad around laser forward direction for the case of a cylindrical target (black solid line) and a planar target (red dashed line).

From our cylindrical filament protons are emitted into a large solid angle, cf. Fig. 2g–i). In Fig. 5a), we show the proton phase-space density at $t=1131\,{\omega }_{{\rm{L}}}^{-1}$ from the simulation. The proton emission is rather uniform within a half-opening angle of 75° with respect to the laser direction, both in terms of proton numbers and spectrum. In particular, the spectral modulation described before is visible by the dark-blue half-ring, which is surrounded by a narrow light-green half-ring, both centered around the origin. In the experiment, the proton beam showed a rather uniform distribution over the area covered by the scintillator in both transverse directions. When assuming a similar opening angle as observed in the simulation, the total conversion efficiency from laser to protons is on the order of 10%, which is quite a high value when compared to results reported so far from similar laser systems but using different targets. Simulations performed at higher laser energies (15 J instead of 2.5 J), but with otherwise similar laser and target parameters showed a shift of the maximum proton energy and the spectral dip to 45 MeV and 28 MeV, respectively. This is an increase by a factor of 4 compared to the low-energy simulation shown in Fig. 4a). Since in our experiment the protons are initially accelerated by TNSA, this scaling agrees with our interpretation. When assuming a similar scaling for the conversion efficiency with laser energy as reported by Robson *et al*.^12^, the conversion efficiency will increase well beyond 20% for 10-J-class laser systems, significantly more than what is achievable with any other type of target so far.

Additionally, we performed simulations with a rectangular target profile (1 × 20 *μ*m^2^). While the proton spectra are comparable to the cylindrical case, their phase space is remarkably different, cf. Fig. 5b). The proton emission half angle is reduced to 14°. The proton energy spectra are compared in Fig. 5c) for the case of the cylindrical (solid black line) and the planar target (dashed red line). Even though the conversion efficiency reduces by 30% due to the larger plasma slab size, the proton numbers within this emission angle are higher by a factor of 8.6 as compared to the cylindrical filament. Such target cross sections could be realized using a rectangular nozzle.

In conclusion, our results show that cryogenic solid hydrogen targets are very promising candidates for optimizing laser-based proton sources for future applications, for which a high conversion efficiency is a requirement. While the protons are emitted under a large opening angle, the energy conversion efficiency for our solid hydrogen target in combination with high-repetition rate lasers is higher than for any other solid or gaseous targets used so far. Using solid hydrogen targets with a rectangular cross-section should allow tailoring of the proton beam profile making laser-accelerated protons from solid hydrogen targets a promising source for applications.
